# Increased plasma soluble endoglin levels as an indicator of cardiovascular alterations in hypertensive and diabetic patients

**DOI:** 10.1186/1741-7015-8-86

**Published:** 2010-12-20

**Authors:** Ana M Blázquez-Medela, Luis García-Ortiz, Manuel A Gómez-Marcos, José I Recio-Rodríguez, Angel Sánchez-Rodríguez, José M López-Novoa, Carlos Martínez-Salgado

**Affiliations:** 1 Unidad de Fisiopatología Renal y Cardiovascular, Instituto Reina Sofía de Investigación Nefrológica, Universidad de Salamanca, Salamanca, Spain; 2 Unidad de Investigación, Centro de Salud La Alamedilla, SACYL, Salamanca, Spain; 3 Servicio de Medicina Interna, Hospital Universitario de Salamanca, Salamanca, Spain; 4 Instituto de Estudios de Ciencias de la Salud de Castilla y León (IECSCYL), Unidad de Investigación, Hospital Universitario de Salamanca, Paseo San Vicente 58-182, 37007 Salamanca, Spain

## Abstract

**Background:**

Endoglin is involved in the regulation of endothelial function, but there are no studies concerning its relation with hypertension- and diabetes-associated pathologies. Thus, we studied the relationship between plasma levels of soluble endoglin and cardiovascular alterations associated with hypertension and diabetes.

**Methods:**

We analyzed 288 patients: 64 with type 2 diabetes, 159 with hypertension and 65 healthy patients. We assessed the relationship of soluble endoglin plasma levels measured by enzyme-linked immunosorbent assay with basal glycemia, glycosylated hemoglobin, blood pressure, endothelial dysfunction (assessed by pressure wave velocity), hypertensive retinopathy (by Keith-Wagener classification), left ventricular hypertrophy (by Cornell and Sokolow indexes), cardiovascular risk and target organ (heart, vascular, kidney) damage.

**Results:**

There are significant correlations between endoglin and glycemia, systolic blood pressure, pulse pressure, pressure wave velocity and electrocardiographically assessed left ventricular hypertrophy. Endoglin levels were significantly higher in patients with diabetes who had nondipper and extreme dipper circadian blood pressure patterns than in dipper circadian patterns, in patients with hypertension and diabetes who had riser pattern than in the other patients, and in patients with diabetes but not hypertension who had extreme dipper pattern than in dipper, nondipper and riser groups. There was also a significant correlation between plasma-soluble endoglin and lower levels of systolic night-day ratio. Higher endoglin levels were found in patients with diabetes who had retinopathy, in patients with diabetes who had a high probability of 10-year cardiovascular risk, and in patients with diabetes and hypertension who had three or more damaged target organs (heart, vessels, kidney) than in those with no organs affected.

**Conclusions:**

This study shows that endoglin is an indicator of hypertension- and diabetes-associated vascular pathologies as endothelial dysfunction and cardiovascular damage.

## Background

Vascular disease is the main cause for disability and death in patients with diabetes mellitus [1]. In type 2 diabetes, small vessels (microangiopathy) or large vessels (macroangiopathy) are affected. Microvascular disease is characteristic of several pathologies such as retinopathy, nephropathy, neuropathy and impaired wound healing, whereas diabetic macroangiopathy is detected by accelerated atherosclerosis and damages vital organs such as heart and brain, being responsible for the increased risk of myocardial infarction, stroke and lower-limb amputation [2,3]. The initial lesion of atherosclerosis involves changes in the vascular endothelium [4], and patients with diabetes invariably show an impairment of endothelium-dependent vasodilation, which is partly due to the frequent association of the disease with other cardiovascular risk factors, including hypertension, obesity and dyslipidemia [5]. The term *endothelial dysfunction *refers to a condition in which the endothelium loses its physiological properties and shifts toward a vasoconstrictor, prothrombotic and proinflammatory state [3]. The fundamental feature of this condition is the impaired nitric oxide (NO) bioavailability that can be the consequence of either a reduced production by endothelial nitric oxide synthase (eNOS) or an increased removal by reactive oxygen species [6]. Hyperglycemia, insulin resistance, hyperinsulinemia and dyslipidemia independently contribute to endothelial dysfunction through different mechanisms, but increased oxidative stress seems to be the first alteration triggering several others [3].

Endoglin (also known as CD105), a type I transmembrane glycoprotein highly expressed on proliferating vascular endothelial cells (ECs) [7], has been identified as an accessory receptor for transforming growth factor-β (TGF-β) [8]. It is expressed as a 180-kDa homodimer formed by disulfide-linked monomers [9]. The human endoglin gene has been localized to chromosome 9q34ter [10], and it is mutated in the Rendu-Osler-Weber syndrome or hereditary hemorrhagic telangiectasia type 1 (HHT1) [11]. Mice lacking endoglin die during the embrionary phase due to defective angiogenesis [12], and endoglin plays a major role in tumoral and nontumoral adult angiogenesis [13,14]. A soluble form of endoglin also plays a central role in preeclampsia, a disease characterized by hypertension and severe alterations in placental circulation [15]. Overall, these data support the view that endoglin has a pivotal function in vascular development and disease [13].

Endoglin is expressed at low levels in resting ECs, but it is highly expressed in vascular ECs during embryogenesis [16], in inflamed tissues and healing wounds [17], psoriatic skin [18], inflamed synovial arthritis [19], upon vascular injury [20] and in tumor vessels [7,13] and in the proliferating endothelium of tissues undergoing angiogenesis [13]. Endoglin is expressed in ECs and in several other cell types involved in the cardiovascular system. For example, while endoglin expression is low in normal smooth muscle cells [21], its expression is upregulated in vascular smooth muscle cells of human atherosclerotic plaques [22]. Endoglin is also expressed in cardiac fibroblasts and modulates the profibrogenic actions of angiotensin II [23].

A soluble form of endoglin (Sol-endoglin) has been detected in plasma, serum and urine from patients with pathologies such as preeclampsia and cancer, and its peptide sequence suggests that it is an N-terminal cleavage product of full-length, membrane-bound endoglin [15]. Uterine ischemia and/or hypoxia play a major role in increased Sol-endoglin release [24]. In addition to being a reliable biomarker of the disease, it has been suggested that Sol-endoglin plays a major role as an antiangiogenic factor in preeclampsia [25]. A possible mechanism involved in the antiangiogenic effects of Sol-endoglin is based on its inhibitory effect on TGF-β1-mediated eNOS activation in ECs [15]. *In vitro *studies have demonstrated that Sol-endoglin impairs EC proliferation and capillary formation [15]. Sol-endoglin also seems to be a regulator of vascular tone, as administration of Sol-endoglin to mice induces an increase in arterial pressure by increasing vascular resistance [15]. Thus, Sol-endoglin seems to impair endothelial function, and endothelial dysfunction is a major characteristic of patients with diabetes.

We have assessed the relationship between plasma levels of Sol-endoglin and vascular alterations associated with diabetes, specifically blood pressure, endothelial dysfunction, retinopathy, degree of left ventricular hypertrophy, cardiovascular risk and target organ damage in patients with type 2 diabetes and hypertension compared to control patients of similar age.

## Methods

This is a cross-sectional study performed in patients with diabetes with or without hypertension. Patients enrolled in the study over a period of 24 months (from January 2008 to January 2010) were from the Internal Medicine Section, University Hospital of Salamanca, and from the Primary Care Research Unit of La Alamedilla Health Centre (Castilla y León Health Service-SACYL), Salamanca, Spain, which covers a population of 46,000 inhabitants. Altogether, 288 consecutive patients complied with the inclusion or exclusion criteria and were asked to participate in the study. Groups of patients were as follows: 64 patients with diabetes (42 of them were hypertensive) and 159 patients with hypertension but not diabetes. Sixty-five healthy individuals were selected as a control group.

Inclusion criteria were patients diagnosed with diabetes mellitus 2 and/or hypertension and aged 20-80 years, with none of the following exclusion criteria: patients unable to comply with the protocol requirements (psychological and/or cognitive disorders, failure to cooperate, educational limitations and problems in understanding written language, and failure to sign the informed consent document), patients participating or who were going to participate in a clinical trial during the study, and patients with serious comorbidities representing a threat to life. Most of the patients with hypertension and diabetes received drug therapy (except those controlled by diet), which is described in Table 1.

**Table 1:** Drug therapies administered in patients with hypertension and/or diabetes

	Total	DIA without HYP	DIA with HYP	HYP without DIA
Antihypertensive drugs (%)	47.21		100.00	58.19
Diuretics (%)	41.67		50.00	43.26
ACEi (%)	39.58		43.75	33.65
ARB (%)	34.03		40.00	35.58
β-blockers (%)	29.86		31.25	32.69
α-blockers (%)	5.56		7.50	2.88
Calcium antagonists (%)	18.06		23.75	18.27
Other antihypertensive drugs (%)	1.39		0.00	1.92
Antidiabetic drugs (%)	21.987	84.38	87.50	
Insulin (%)	20.90	29.63	20.00	
Metformin (%)	83.58	85.19	80.00	
Sulfonylureas (%)	32.84	14.81	37.14	
Meglitinides (%)	2.99	7.41	4.29	
α-glucosidase inhibitors (%)	1.49		1.43	
Glitazones (%)			7.14	

Data are expressed as percentages. ACEi, angiotensin-converting enzyme inhibitors; ARB, angiotensin receptor blockers; DIA, patients with diabetes; HYP, patients with hypertension.

Hypertension was diagnosed when the mean of three separate measurements of blood pressure over time was ≥ 140 mmHg for systolic blood pressure (SBP) and/or ≥ 90 mmHg for diastolic blood pressure (DBP). At each measurement, blood pressure was measured at least twice, separated by more than 1 minute, as recommended by The Task Force for the Management of Arterial Hypertension of the European Society of Hypertension and of the European Society of Cardiology [26]. Diabetes was diagnosed when fasting glucose level was ≥ 126 mg/dL or ≥ 200 mg/dL 2 hours after oral glucose overload (repeated on two occasions) or after detection of symptoms of diabetes and random blood glucose ≥ 200 mg/dL as recommended by the Expert Committee on the Diagnosis and Classification of Diabetes Mellitus [27].

### Ethical and legal issues

The experimental protocol was in accordance with the Declaration of Helsinki (2000) of the World Medical Association and also was in agreement with the guidelines of and approved by the Ethics Committee of the University Hospital of Salamanca, Spain, and complied with Spanish data protection law 15/1999 and its developed specifications (RD 1720/2007). Each patient included in the study signed an informed consent form to participate in the study after full explanation of the purpose and nature of all procedures used. To guarantee data confidentiality, all the electronic and paper copies of the protocol, signed informed consent documents and results of the tests were kept locked in a safe place, and only the study investigators had access to the data on the people who agreed to participate in the study.

### Sociodemographic and cardiovascular variables

We evaluated the next sociodemographic variables and cardiovascular risk factors: patient age and sex, hypertension, dyslipidemia, alcohol consumption, smoking, physical activity and history of premature cardiovascular disease (before 55 years of age in males and before 65 in females) in first-degree relatives, myocardial infarction, angina, revascularization, heart failure, atrial fibrillation and cerebrovascular events (ischemic stroke, intracranial haemorrhage and transient brain ischemia), as well as the presence of symptomatic peripheral arterial disease.

### Anthropometric measurements

Body weight was determined at two different times using a homologated electronic scale (Seca 770; Seca, Hamburg, Germany) following due calibration (precision ± 0.1 kg), with the patient wearing light clothing and without shoes. Readings were rounded to 100 g. Height was measured with a portable system (Seca 222), recording the average of two readings, and with the patient shoeless in the standing position. The values were rounded to the closest centimeter. Body mass index (BMI; measured in kilograms per meter squared) was also calculated. Waist circumference was measured using a flexible, graduated measuring tape with the patient in the standing position without clothing. The upper border of the iliac crests was located, and the tape was wrapped just above this point without compressing the skin. The reading was taken at the end of a normal breath according to the recommendations of the 2007 SEEDO Conference [28].

### Biochemical determinations

Blood samples were collected in the morning, after the patient had fasted for at least 8 hours prior to other measurements. Physiological determinations were creatinine, basal glucose and glycosylated hemoglobin (HbA1c), high-density lipoprotein (HDL) cholesterol, low-density lipoprotein (LDL) cholesterol, total cholesterol and triglycerides in blood and microalbuminuria. The parameters were measured on a blind basis in a General Hospital Biochemistry laboratory using standard authomatized techniques.

### Blood pressure determination

Office blood pressure evaluation involved three measurements of SBP and DBP using the average of the last two measurements, with a validated OMRON model M7 sphygmomanometer (Omron Health Care, Kyoto, Japan), following the recommendations of the European Society of Hypertension [29]. Pulse pressure was estimated with the mean values of the second and third measurements.

Home blood pressure was self-measured using an OMROM model M7 sphygmomanometer as previously described [30]. Patients were apprised of how to perform blood pressure recordings at home, and educational leaflets were developed to ensure that the patients correctly performed these self-measurements. A self-registry sheet was provided to ensure correct pressure recording.

Ambulatory blood pressure monitoring was performed on a standard activity day, with a SpaceLabs 90207 model (Spacelabs Healthcare, Issaquah WA, USA), validated according to the protocol of the British Hypertension Society [31]. Records in which the percentage of valid readings was ≥ 66% of the total measurements were considered to be valid. Furthermore, a 24-hour ambulatory blood pressure monitoring device was fitted. At least 14 measurements (every 20 min) during the daytime or at least 7 measurements (every 30 min) during the nighttime or resting period were required. The average and dispersion estimators of SBP and DBP were calculated along 24 h, including daytime and nighttime periods, on the basis of the diary reported by the patient, in which bedtime and wakeup time were specified.

### Evaluation of peripheral artery disease

Peripheral artery disease (PAD) was evaluated in the morning using the ankle-brachial index (ABI), with the patient having refrained from consuming caffeine-containing beverages and alcohol, and not having smoked, in the previous 12 h, and with room temperature ranging between 22°C and 24°C. With the feet uncovered and in a supine decubitus position after resting for 20 min, pressure in the lower extremities was measured using a portable Doppler system Minidop Es-100Vx (Hadeco Inc., Miyamae-ku Kawasaki, Japan) applying the probe at the anterior or posterior tibial artery at an angle of approximately 60° to blood flow direction. Blood pressure was also measured twice at 3- to 5-minute intervals in both arms. ABI was calculated as previously described [32]. Subclinical PAD was diagnosed if ABI was lower than 0.9 [33].

### Identification of left ventricular hypertrophy

Electrocardiographic (ECG) examination was performed with a General Electric MAC 3.500 ECG System (Niskayuna NY, USA), which measures voltage and duration of waves and estimates the criteria of the Cornell voltage duration product (Cornell VDP) [34] to assess left ventricular hypertrophy (LVH) using the following equations: (RaVL + SV3) × QRS in men and (RaVL + SV3) × QRS + 6 in women. LVH was defined as the VDP value greater than 2,440 mV/ms [26]. LVH was also determined as the next voltage sum: S wave in V1 + R wave in lead V5 or V6 ≥ 35 mm using Sokolow-Lyon voltage criteria [35].

### Renal function assessment

Kidney damage was assessed by measuring plasma creatinine concentrations, glomerular filtration rate was estimated by CKD-EPI (Chronic Kidney Disease Epidemiology Collaboration) [36] and the MDRD-IDMS (Modification of Diet in Renal Disease-Isotopic Dilution Mass Spectrometry) [37] formulas, and proteinuria was assessed using the albumin-creatinine ratio following the 2007 European Society of Hypertension/European Society of Cardiology Guidelines criteria [26]. Subclinical organ damage was defined as plasma creatinine between 1.3 and 1.5 mg/dL in men and 1.2 and 1.4 mg/dL in women, glomerular filtration rate below 60 ml/min or albumin-creatinine ratio > 22 mg/g in men and 31 mg/g in women. Renal disease was defined as plasma creatinine of 1.5 mg/dL or higher in men or 1.4 mg/L in women or albumin-creatinine ratio > 300 mg/24 h.

### Evaluation of retinopathy

Retinography was performed with a Topcon TRC NW 200 nonmydriatic retinal camera (Topcon Europe B.C., Capelle a/d Ijssel, The Netherlands), obtaining images centered on the papilla. Captured images were classified by two independent observers according to the Keith-Wagener classification for hypertensive retinopathy [38]. Retinopathy was classified as grade I (generalized arteriolar constriction), grade II (irregularly located, tight constrictions), grade III (retinal edema, cotton-wool spots and flame haemorrhages, sclerosis and spastic lesion of arterioles, hard exudates including a macular star) and grade IV (same as grade III but with swelling of the optic disk and silver wiring). Grade III or IV was considered to be associated with cardiovascular disease [26].

### Determination of endothelial dysfunction by evaluation of pulse wave velocity

Pulse wave velocity (PWV) was calculated in the morning (between 8:30 and 11:00 AM) 1-2 days before blood was drawn, using the SphygmoCor System (Vx Pulse Wave Velocity; AtCor Medical, West Ryde, Australia) with patients in supine decubitus position [39]. This analysis was performed in a blinded fashion by the same trained researcher who was completely unaware of the health status of the patients analyzed. Carotid and femoral pulse waves were analyzed, estimating the delay in the ECG wave and calculating the PWV. Space measurements were taken with a measuring tape from the suprasternal notch to the carotid and femoral arteries at the sensor location. A measurement of PWV higher than 12 m/s was considered endothelial dysfunction [26].

Pulse wave analysis was performed with a sensor in the radial artery using mathematical transformations to estimate the aortic pulse wave with the SphygmoCor system (Px Pulse Wave Analysis), with patients in sitting position and resting the arm on a rigid surface. The aortic wave morphology was used to estimate central (aortic) arterial pressure along with central ventricular load, diastolic perfusion pressure, subendocardial viability index, pressure increment, central pulse pressure and the augmentation index (PWA), defined as the percentage of the increase in central pulse pressure: AIx = pressure increase × 100/pulse pressure [40].

### Cardiovascular risk assessment

Cardiovascular risk of patients was assessed using the scale of the ESH-ESC guide 2007 [26]. Risk categories were average risk (adjusted for age and sex), low added risk, moderate, high or very high risk.

### Plasma soluble endoglin determination

Sol-endoglin concentration in plasma samples was evaluated using an enzyme-linked immunosorbent assay method (Human Endoglin; R&D Systems, Minneapolis, MN, USA) following the instructions of the manufacturer. With regard to specificity, the assay recognizes recombinant and human endoglin without significant cross-reactivity or interference with other members of the TGF-β family. With regard to sensitivity, the minimum detectable dose ranged from 0.001 to 0.030 ng/mL. Absorbance (proportional to the initial amount of endoglin) was determined using a spectrophotometer (Thermo Luminoscan Ascent, Waltham, MA, USA) at 450 nm with a wavelength correction of 540 nm.

### Statistical analysis

Data input was performed using the Teleform system (Autonomy Cardiff, Vista, CA, USA) and exporting the data to the PASW version 18.0 statistical package (SPSS Inc., Chicago, IL, USA) for data analysis. Data followed a normal distribution as confirmed by kurtosis normality of residuals test. Data were presented as means ± standard error of the mean (SEM) or standard deviation in the case of quantitative variables, and as frequency distributions for qualitative variables. The χ^2 ^test was used to analyze associations between qualitative variables. Student's *t*-test was used for independent samples to compare quantitative variables for two groups, and one-way analysis of variance was used for more than two groups. Pearson's correlation test was used to analyze associations between quantitative variables. A *P *value lower than 0.05 was considered statistically significant. Owing to our sample size (288 patients), our low variability and the selected α value (0.05), the power of our study is 0.95 (β value, 0.05).

## Results

General and medical characteristics of the patients are presented in Table 2; 46.23% of patients presented overweight (BMI > 25 kg/m^2^) and 32.13% of patients were obese (BMI > 30 kg/m^2^) (Table 2).

**Table 2:** Demographic, physical and medical characteristics of the patients included in the study

	Total	DIA without HYP	DIA with HYP	HYP without DIA	Controls
Number	288	22	42	159	65
Age (yr)	54.90 ± 11.68	53.27 ± 12.74	62.17 ± 8.03	56.69 ± 11.08	48.75 ± 11.22
Male sex (%)	62.29	81.81^a^	66.67^a^	62.78	53.85
Body mass index (kg/m2)	28.32 ± 4.26	28.71 ± 4.99	30.33 ± 4.86	28.68 ± 3.93	26.82 ± 3.29
Endoglin (ng/mL)	5.04 ± 1.05	5.02 ± 0.98	4.88 ± 1.20	4.39 ± 1.04	5.21 ± 1.10
Basal glycemia (mg/dL)	98.76 ± 30.77	121.22 ± 30.03^b,e^	132.56 ± 45.52^b,e^	88.45 ± 11.49	84.47 ± 9.04
HbA1c (%)	5.42 ± 1.05	6.72 ± 1.07^b,e^	6.80 ± 1.30^b,e^	5.03 ± 0.45	4.91 ± 0.45
Systolic blood pressure (mmHg)	125.89 ± 12.72	121.34 ± 8.37	126.20 ± 14.21^c^	127.03 ± 13.94^c^	123.69 ± 9.52
Diastolic blood pressure (mmHg)	77.05 ± 9.43	73.38 ± 6.08	72.78 ± 9.11^a,d^	76.82 ± 9.88^c^	77.65 ± 7.65
Heart rate (beats/min)	71.96 ± 10.56	75.11 ± 11.29	72.51 ± 10.28	71.08 ± 11.06^c^	72.77 ± 10.31
Leukocytes (10^3 ^cells/μL)	6.70 ± 1.95	7.35 ± 2.90^a^	7.23 ± 1.94^a^	6.71 ± 1.96^a^	6.08 ± 1.03
Neutrophils (10^3 ^cells/μL)	3.52 ± 1.33	4.06 ± 2.30^a^	3.76 ± 1.32^a^	3.52 ± 1.25	3.17 ± 0.93
Lymphocytes (10^3 ^cells/μL)	2.41 ± 0.74	2.49 ± 0.61	2.71 ± 0.77	2.42 ± 0.83	2.23 ± 0.52
Monocytes (10^3 ^cells/μL)	0.54 ± 0.26	0.56 ± 0.19	0.58 ± 1.18	0.55 ± 0.32	0.47 ± 0.15
Eosinophils (10^3 ^cells/μL)	0.21 ± 0.22	0.19 ± 0.10	0.24 ± 0.17	0.23 ± 0.28	0.18 ± 0.11
Basophils (10^3 ^cells/μL)	0.04 ± 0.03	0.04 ± 0.02	0.03 ± 0.02	0.04 ± 0.03	0.03 ± 0.03
Smokers (%)	24.92	36.36	16.67	26.81	24.61
High-density lipoproteins (mg/dL)	52.35 ± 12.42	46.27 ± 8.85^a,d^	47.43 ± 9.74^a,d^	53.50 ± 12.41	54.13 ± 13.51
Low-density lipoproteins (mg/dL)	125.80 ± 32.67	113.72 ± 27.18^a^	105.63 ± 25.52^a,d^	131.43 ± 34.14^c^	127.13 ± 28.74
Alcohol consumption (U/wk)	11.30 ± 20.33	9.79 ± 15.82	12.70 ± 20.94	12.21 ± 23.45	10.15 ± 18.06
Daily physical activity (%)	34.75	31.82	57.14^a^	34.78	26.15
FPACD (%)	15.08	9.09	30.95^a,c,d^	13.77	9.23

Data are expressed as means ± standard deviation or percentage. DIA, patients with diabetes; HbA1c, glycosylated hemoglobin; HYP, patients with hypertension; FPACD, family premature antecedents of cardiovascular disease in first-degree relatives. ^a^Statistically significant differences (*P *< 0.05) vs. control group; ^b^*P *< 0.0001 vs. control group; ^c^*P *< 0.05 vs. DIA without HYP; ^d^*P *< 0.05 vs. HYP without DIA; ^e^*P *< 0.0001 vs. HYP without DIA.

### Endoglin and diabetes

In our study population, there was a positive correlation between Sol-endoglin plasma levels and basal glycemia in patients with diabetes and hypertension and between endoglin levels and glycated hemoglobin in all patients with diabetes (Table 3).

**Table 3:** Pearson's correlations between plasma endoglin and basal glycemia, glycated hemoglobin and cardiovascular parameters

	All DIA R P value	DIA without HYP R P value	DIA with HYP R P value	All HYP R P value	HYP without DIA R P value	Control R P value
Basal glycemia	0.221 0.079	-0.230 0.304	0.360 0.019^a^	0.065 0.388	-0.130 0.128	-0.095 0.451
Glycated hemoglobin	0.257 0.042^a^	0.347 0.114	0.239 0.132	0.008 0.912	-0.118 0.167	-0.097 0.451
Systolic blood pressure	0.243 0.053	-0.072 0.751	0.381 0.013^a^	0.183 0.031^a^	0.009 0.918	-0.076 0.547
Pulse pressure	0.326 0.009^a^	-0.150 0.506	0.519 0.000^a^	0.118 0.115	-0.024 0.784	0.064 0.611
Systolic night-day ratio	-0.109 0.391	-0.481 0.019^a^	0.037 0.817	-0.033 0.665	-0.063 0.468	0.009 0.946
Heart rate	0.053 0.678	0.508 0.016^a^	-0.141 0.374	-0.054 0.474	-0.023 0.788	-0.232 0.064
Pulse wave velocity	0.194 0.127	-0.131 0.562	0.348 0.026^a^	0.009 0.905	-0.096 0.262	-0.144 0.251
VDP Cornell	0.382 0.002^a^	0.273 0.219	0.439 0.004^a^	0.148 0.047^a^	0.027 0.752	-0.171 0.174
Sokolow index	0.223 0.076	0.127 0.575	0.294 0.058	0.151 0.043^a^	0.093 0.281	0.071 0.575

DIA, diabetic patients; HYP, hypertension; R, correlation coefficient; VDP, voltage-duration product. ^a^Statistically significant differences (*P *< 0.05).

### Endoglin and blood pressure

**Figure 1:**
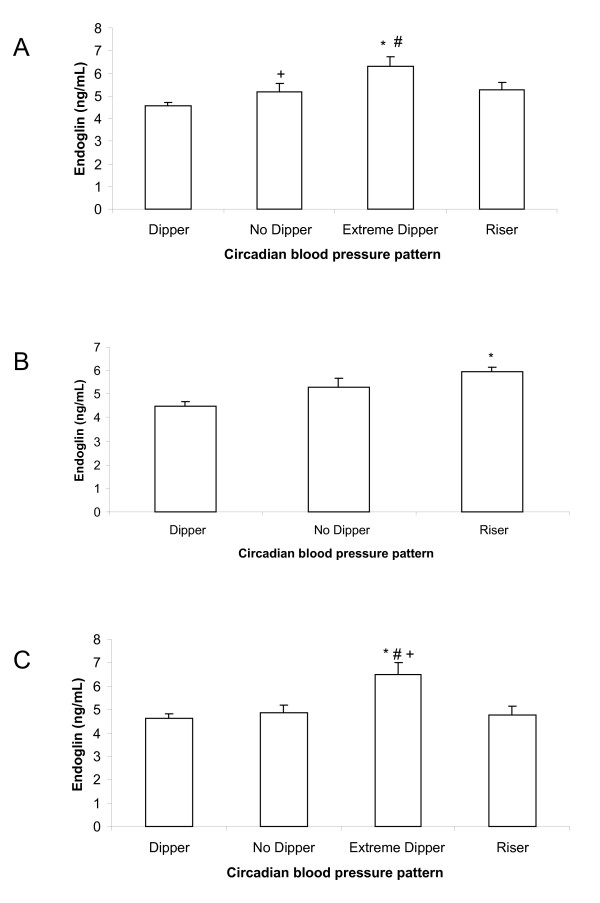
**Comparison of Sol-endoglin plasma levels and circadian blood pressure pattern**. **(A) **Patients with diabetes. **(B) **Patients with hypertension and diabetes. **(C) **Nonhypertensive patients with diabetes. Data (ng/mL) are expressed as means ± SEM. One-way analysis of variance (ANOVA): *P < 0.01 **(A and C) **and *P *< 0.05 **(B) **vs. dipper; #*P *< 0.05 vs. nondipper; +*P *< 0.05 vs. dipper **(A) **and vs. riser **(C)**.

There was a significant correlation between plasma Sol-endoglin concentration and SBP (in both office evaluation and home self-measurement) in patients with diabetes and hypertension and in all patients with hypertension with or without diabetes (Table 3). There was also a strong correlation between pulse pressure and Sol-endoglin plasma levels in patients with diabetes, either with or without hypertension (Table 3). Furthermore, endoglin levels were different depending on the circadian blood pressure pattern. Thus, endoglin levels were higher in all patients with diabetes with nondipper (absence of the normal nocturnal fall in blood pressure) and extreme dipper (with marked nocturnal blood pressure falls) than in dipper circadian patterns (Figure 1A). Endoglin levels were also higher in patients with hypertension and diabetes with riser circadian pattern (nocturnal blood pressure increase compared with daytime blood pressure) than in the other patients (Figure 1B). Moreover, nonhypertensive patients with diabetes with extreme dipper pattern showed higher plasma levels of Sol-endoglin than did dipper, nondipper and riser groups (Figure 1C), and endoglin showed a negative correlation with systolic night-day ratio in these patients (Table 3). On the other hand, we also observed a positive correlation between heart rate and plasma Sol-endoglin in nonhypertensive patients with diabetes (Table 3).

### Endoglin and endothelial dysfunction

Endothelial dysfunction was evaluated by the analysis of PWV, which indicates arterial stiffness. Our study show that plasma Sol-endoglin levels showed a positive correlation with PWV values in patients with hypertension and diabetes (Table 3).

### Endoglin and retinopathy

**Figure 2:**
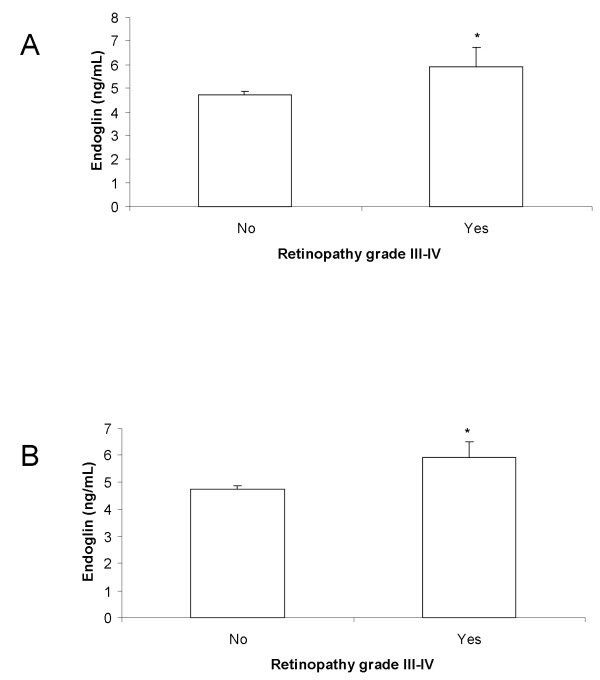
**Comparison of Sol-endoglin plasma levels and Keith-Wagener retinal grades III and IV classification**. **(A) **Patients with hypertension and diabetes. **(B) **All patients with diabetes. Data (ng/mL) are expressed as means ± SEM. One-way ANOVA: *P < 0.05 **(A) **and *P *< 0.01 **(B)**.

We found a strong relation between Sol-endoglin plasma levels and retinopathy. Higher endoglin levels (above 5 ng/mL) were found in all patients with diabetes (odds ratio, 4.72) and in patients with hypertension and diabetes (odds ratio, 3.54) categorized as grade III and grade IV in the Keith-Wagener retinal changes classification, in both right and left eyes compared with patients with grades I and II (Figure 2).

### Endoglin and left ventricular hypertrophy

LVH was evaluated by electrocardiography using both Cornell and Sokolow indexes. We observed positive correlations between plasma Sol-endoglin levels and Cornell-VDP in patients with hypertension as well as in all patients with diabetes (Table 3). There was also a positive correlation between endoglin levels and Sokolow index in patients with hypertension (Table 3).

### Endoglin, cardiovascular risk and target organs damage

**Figure 3:**
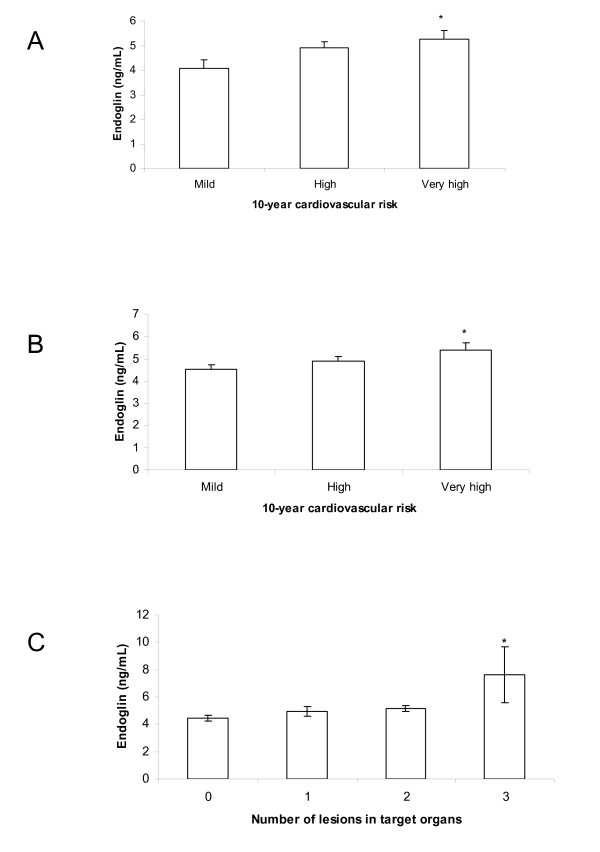
**Comparison of Sol-endoglin plasma levels, 10-year cardiovascular risk and number of damaged target organs**. **(A) **10-year cardiovascular risk in patients with hypertension and diabetes. **(B) **All patients with diabetes. **(C) **Number of damaged target organs in patients with patients hypertension and diabetes. Data (ng/mL) are expressed as means ± SEM. One-way ANOVA: *P < 0.05 vs. mild group **(A and B) **and vs. 0 group **(C)**.

We observed that plasma Sol-endoglin levels were higher in patients with hypertension and diabetes and in all patients with diabetes with very high cardiovascular risk in the next 10 years compared with patients with mild vascular risk. Although we have not found any significant relationship or correlation between Sol-endoglin and renal dysfunction, endoglin levels were higher in patients with diabetes with three or more target organs damaged than in those with no organs affected (Figure 3).

## Discussion

Our data shows that Sol-endoglin plasma levels are significantly related to glycemia, SBP, pulse pressure, PWV, heart rate and the degree of LVH assessed by ECG. Sol-endoglin plasma levels are different in patient groups depending on the circadian blood pressure pattern, and increased endoglin levels are associated with retinopathy, very high 10-year cardiovascular risk and increased number of damaged target organs in patients with type 2 diabetes. This is the first study in humans showing that plasma Sol-endoglin concentration could serve as an indicator of diabetes-associated pathologies such as hypertension, endothelial dysfunction and cardiovascular risk. It should be noted that a high correlation coefficient has been obtained between plasma Sol-endoglin levels and heart rate (0.508; *P *= 0.016), and this is especially important as heart rate has been recently suggested to be a marker of increased vascular risk [41].

Sol-endoglin seems to be a regulator of vascular tone. We show that Sol-endoglin levels are higher in patients with diabetes who have increased levels of SBP; according to our findings, in an experimental model with pregnant rats, Gilbert *et al*. [24] showed that placental ischemia increases the expression of Sol-endoglin and provokes hypertension, thus mimicking the pathophysiologic features of preeclampsia. Moreover, overexpression of Sol-endoglin in rodents was found to induce hypertension and increased vascular permeability [42]. It has been shown that injection of Sol-endoglin into rats induced hypertension [15]. Recent studies from our group have shown that transgenic mice overexpressing human Sol-endoglin are hypertensive (unpublished results). To explain the mechanism involved, it has been proposed that Sol-endoglin induces its prohypertensive effects through interaction with circulating endoglin-binding molecules, such as the TGF-β protein superfamily, thus preventing the binding of these molecules to the TGF-β receptor complex [25].

The relevance of endoglin in the cardiovascular system is reflected by the fact that mutations in the endoglin gene cause a vascular disease called the Rendu-Osler-Weber syndrome or HHT1 [11], which is characterized by vascular dysplasia, frequent episodes of epistaxis, mucocutaneous telangiectases and arteriovenous malformations of the lung, brain, liver and gastrointestinal tract [43]. A reduction in Sol-endoglin levels has been recently described in patients with HHT1 [44]. About 15%-35% of HHT patients show pulmonary arteriovenous malformations, 10% have cerebral arteriovenous malformations, 25%-33% suffer significant gastrointestinal blood loss from gastrointestinal tract telangiectasia and an unknown but high percentage have liver involvement. In summary, 10% of HHT patients die prematurely or experience major disability, largely because of bleeding or paradoxical embolization through pulmonary or cerebral arteriovenous malformations [45]. Ocular manifestations include conjunctival, retinal and choroidal telangiectasia [46]. Although it has been recently shown that the loss of endoglin leads to retinal vascular abnormalities in mice [47], the frequency of ocular manifestation in patients with HHT is estimated to be 1.9% for retinal involvement [46]. The prevalence of cardiovascular disease other than that mentioned above in patients with HHT1 does not seem to be higher than in the general population when adjusted by other endoglin-independent risk factors [43]. Nevertheless, the results obtained in our study seem to suggest that S-endoglin levels are associated with cardiovascular damage, which does not mean that they are themselves the cause of such damage.

It has been reported that administration of Sol-endoglin to mice elevates arterial pressure by increasing vascular resistance [15]. Most probably, this effect could be attributed to the inhibitory effect of Sol-endoglin on TGF-β1-mediated eNOS activation in ECs, and it has been suggested that high levels of circulating Sol-endoglin could contribute to the hypertension shown by women with preeclampsia [15].

As previously described, the impairment of endothelium-dependent vasodilation in patients with diabetes is due to the impaired NO bioavailability [6], and endoglin expression and NO regulation are intimately related, as we have demonstrated that endoglin plays a major role in regulating eNOS abundance and NO synthesis regulation based on two different mechanisms. First, endoglin regulates eNOS mRNA expression [48-50]. The second mechanism is the regulation of eNOS protein half-life and eNOS activity [51]. Mice deficient in endoglin (*eng^+/-^*) show a defective vasodilator response to endothelium-dependent vasodilator substances such as acetylcholine or bradykinin [48]. In agreement with these data, *eng^+/- ^*mice show a decreased NO synthesis and a decreased eNOS expression [48,49,51]. TGF-β1 leads to an increased vasodilation in control mice that is severely impaired in *eng^+/- ^*mice, suggesting the involvement of endoglin in the TGF-β-regulated vascular homeostasis [50].

Changes in Sol-endoglin plasma levels have been reported in other pathologies different from preeclampsia, such as atherosclerosis and coronary artery disease [52]. Also, changes of Sol-endoglin plasma levels after an acute myocardial infarct are accurate predictors of acute mortality in these patients [53]. Although there are numerous reports on the possible participation of Sol-endoglin in different diseases, the specific molecular mechanism of action of this soluble form of endoglin in these pathologies remains to be elucidated. In preeclampsia, the origin of Sol-endoglin seems to be the destruction of the placental vessels and/or the shedding of membrane-bound endoglin by specific membrane metalloproteases [25]. In the case of patients with diabetes and/or hypertension, we do not know what the origin of the increased plasma Sol-endoglin levels is.

Limitations of our study are its cross-sectional design, which precludes longitudinal analysis between endoglin, cardiovascular risk factors and subclinical organ damage, and the selection of the study population, since sampling was performed consecutively with pragmatic and broad inclusion criteria, including patients with hypertension with a recent diagnosis or short course of hypertension, patients with diabetes and hyperlipidemia, and many patients receiving drug therapy. This could modify blood pressure levels and therefore limit the validity of some results. Consequently, the heterogeneity of the sample could lead to some limitations when interpreting the results, though it is quite similar to the distribution of the real population of short-course patients with hypertension who have some risk factors and without previous cardiovascular disease.

## Conclusions

Endoglin expression has revealed its prognostic and diagnostic value in other circumstances, being a potential vascular target for antiangiogenic cancer therapy [13]. Our study shows the emerging role of endoglin as an indicator of diabetes-associated vascular pathologies such as hypertension, endothelial dysfunction and cardiovascular risk. While much more work must be done to fully understand the molecular mechanisms that underlie the role of endoglin in these pathologies, this study provides new research strategies for better diagnosis, prognosis and therapy in diabetes-associated endothelial dysfunction. These results are innovative and relevant enough to start prospective studies that will allow us to establish the relative strength of the prediction of cardiovascular risk and the appearance of target organ damage according to the endoglin levels presented by the patient.

## Competing interests

The authors declare that they have no competing interests associated with this paper.

## Authors' contributions

AMB-M performed the plasma Sol-endoglin determinations and statistical analyses and contributed to the manuscript writing. LG-O, MAG-M, JIR-R and AS-R were responsible for the recruitment of patients, acquisition of patients' biological samples and data. LGO, MAG-M and JMLN made substantial contributions to the conception and design of the study and were involved in drafting the protocol, the interpretation of data and the preparation of the manuscript. CM-S was responsible for the integrity of the work as a whole, from inception to the published article. All authors read and approved the final version of the manuscript.

## Pre-publication history

The pre-publication history for this paper can be accessed here:

http://www.biomedcentral.com/1741-7015/8/86/prepub
